# Biological Properties and Biomedical Applications of Pectin and Pectin-Based Composites: A Review

**DOI:** 10.3390/molecules28247974

**Published:** 2023-12-06

**Authors:** Naznin Sultana

**Affiliations:** Texas Undergraduate Medical Academy, Prairie View A&M University, Prairie View, TX 77446, USA; nasultana@pvamu.edu

**Keywords:** pectin, tissue engineering, drug delivery, composites, biomedical applications

## Abstract

Pectin has recently drawn much attention in biomedical applications due to its distinctive chemical and biological properties. Polymers like pectin with cell-instructive properties are attractive natural biomaterials for tissue repair and regeneration. In addition, bioactive pectin and pectin-based composites exhibit improved characteristics to deliver active molecules. Pectin and pectin-based composites serve as interactive matrices or scaffolds by stimulating cell adhesion and cell proliferation and enhancing tissue remodeling by forming an extracellular matrix in vivo. Several bioactive properties, such as immunoregulatory, antibacterial, anti-inflammatory, anti-tumor, and antioxidant activities, contribute to the pectin’s and pectin-based composite’s enhanced applications in tissue engineering and drug delivery systems. Tissue engineering scaffolds containing pectin and pectin-based conjugates or composites demonstrate essential features such as nontoxicity, tunable mechanical properties, biodegradability, and suitable surface properties. The design and fabrication of pectic composites are versatile for tissue engineering and drug delivery applications. This article reviews the promising characteristics of pectin or pectic polysaccharides and pectin-based composites and highlights their potential biomedical applications, focusing on drug delivery and tissue engineering.

## 1. Introduction

Recent studies have gained attention on the natural polymer pectin due to its lower price and biological properties that enable it to be used in various pharmacological and biomedical applications [1]. Various developments of simple and more innovative in vitro and in vivo testing to influence immunity, together with manufacturing, purification, and characterization techniques, have immensely contributed to the ongoing research of pectin and pectin-based composites in the food, healthcare, and cosmetic industries for their low toxicity and therapeutic effects. Commercially available pectin satisfies the required specifications and is approved by several Food and Agriculture Organizations for specific applications. Pectin is commercially obtained from the residual part of the plant materials after the extraction of juice (citrus or apple) and sugar (sugar beet). Pectin is an essential part of the cell wall that is needed for the development of plants. Pectin can be efficiently used for drug delivery and tissue engineering through gel beads or microspheres, 3D scaffolds, and membranes.

**Figure 1:**
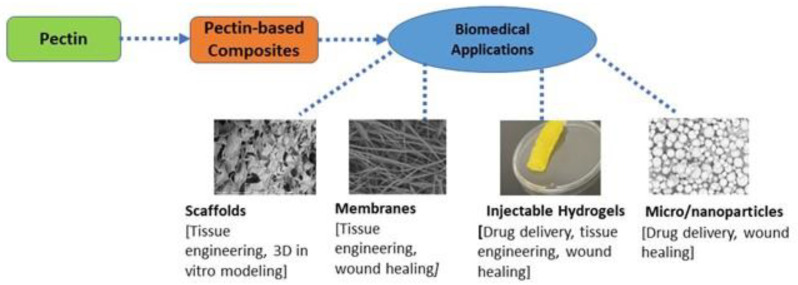
Several biomedical applications of pectin and pectin-based composites.

In all primary cell walls, there exist three significant types of pectic polysaccharides: (i) homogalacturonan, (ii) rhamnogalacturonan-I, and (iii) rhamnogalacturonan-II [2,3,4]—pectin-modifying enzymes and endomembrane system biosynthesis cause the structural complexity and the pectin domains’ heterogeneity. The enzyme pectin methyl esterase can modulate Homogalacturonan. Rhamnogalacturonan-I contains extremely distinct functionally regulated polymers; however, rhamnogalacturonan-II shows a highly stable pectin matrix [2]. Structurally, pectin is classified in a multifunctional family of covalently linked D-galacturonic acid-rich polysaccharides found in terrestrial plants’ primary cell walls [5]. The covalently linked 1-4-alpha-D-galacturonic units are interchangeable with 1-2 attached alpha-L-rhamnopyranosyl remnants that carry saccharide polymers [6]. The galacturonic remnants found in pectin are typically present as salts or methyl esters. The precise chemical structure of pectin is complex to deduce. It depends on the source and conditions they extract in location and other surrounding factors, making their chemical arrangement different. Commercially, pectin is extracted from plant materials such as citrus peel, apple pomace, and sugar beets. The polysaccharide helps to provide intercellular adhesion, rigidity, and mechanical resistance for the cell walls of plants. This support is needed to help plants living in harmful environments related to temperature, pollutants, and other environmental stressors survive. The multifunctional component of pectin has allowed it to provide numerous target sites for chemical modifications [7]. The properties of pectin, such as its nontoxicity, emulsion behavior, diverse chemical composition, biocompatibility, and high stability, enable it to be a commonly used biopolymer. Industrially, pectin is used for various applications such as food manufacturing and biomedical engineering. Biomedical applications of pectin primarily include drug delivery, tissue engineering, and wound healing (Figure 1).

**Figure 2:**
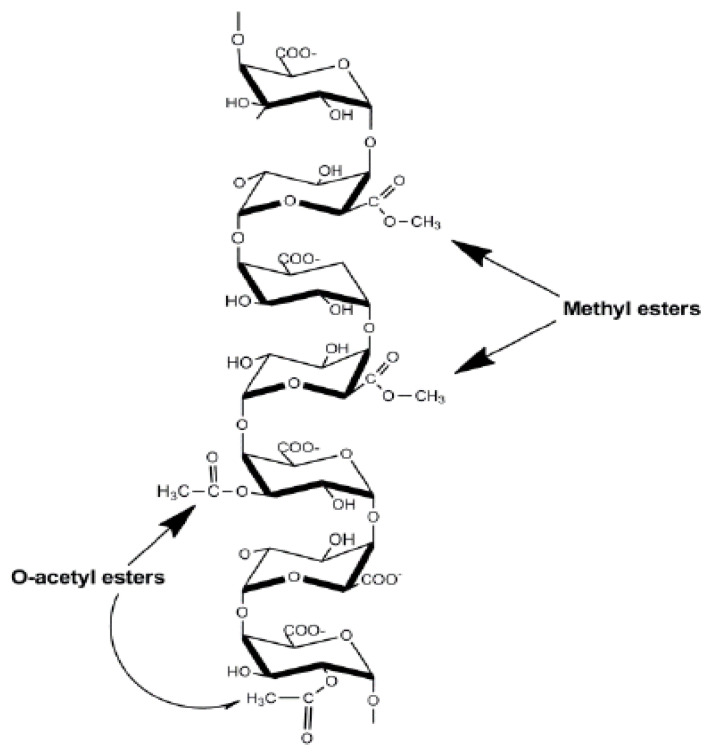
Homogalacturonan structure of pectin polysaccharides. Homogalacturonan is a linear polymer of α-(1,4)-D galacturonic acid with methyl-esterified at C-6 and acetyl-esterified at positions O-2 and O-3 [10].

The structure of pectin differs and depends on the type of plants and cell types that it develops in. Based on the source that the pectin emerges from, the polymer will vary in size, acetylation type, the degree to which it is esterified, and other variables that are controlled by the differences between the galacturonic acid that lead to the homogalacturonan chain and the side chain type of the rhamnogalacturonan-1 [8]. Rhamnogalacturonan-1 generally forms the branched regions of the pectin polysaccharide, which are the primary carbohydrate chains. Interestingly, Homogalacturonan forms the linear fragment of the polysaccharide, and sometimes, the chain forms the component that rhamnogalacturonan-1 generally makes up (Figure 2). The pectin polysaccharide varies based on the source from which it is groomed and the conditions from which it is extracted [9]. Regardless of the diversity found within the pectin polysaccharide, the structure is classified as canonical. Though pectin was discovered over two hundred years ago, the design of its composition has yet to be ultimately interpreted.

The pectin polymer is a core structure alternating alpha-1, 4-linked D-galacturonic acid and alpha-1, 2-L-rhamnose units. The system of pectin regulates the influence that the polysaccharide has on cytokine production; this proves that the elemental characteristics that are found in the polysaccharide are related to its ability to impact cellular environmental conditions. The diversity in the structure of pectin polymers from different plant origins enables it to be used in multiple applications. Pectin extracted from various sources of plants generally has similar structural characteristics, but the structures ultimately differ based on the species and the plant’s physiological stage. With the effect of structural features, the chemical composition of pectin, such as the galacturonic acid proportion, methyl group content, and grade of acetylation, determines the polymer’s function [1].

Immune reactivity is a factor that influences the use of pectin within biomedical and tissue engineering/drug delivery applications [11,12,13]. In applications for pathological conditions, immunomodulators are essential to regulate the body’s distinctive immune response to foreign materials and antigens from foreign or transplanted cells. The purpose of utilizing immunomodulators such as pectin is not to eliminate the immune response but to regulate the reactivity and further the efficiency of the applications that require the modulation of the immune system. Past studies have reported that pectin can weaken inflammatory reactivity by stimulating anti-inflammatory cytokines and decreasing the assembly of proinflammatory cytokines [14].

Due to its structural complexity and diversity, pectin has many applications. Pectin consists of many active functional groups of polysaccharides, enabling them to have much more excellent modification properties than other biopolymers. Pectin is a hydrophilic natural polymer that can absorb or retain much water and exhibit swelling properties. Hydrogels and composite materials can be formed by crosslinking and other techniques, and the matrix structure can be incorporated with various bioactive compounds. Pectin-based smart composites with physical-sensitive (light, temperature, electricity), chemical-sensitive (pH, redox, glucose), and biological-sensitive (enzymes) properties are suitable in the delivery system of bioactive compounds in addition to their suitable biodegradable and biocompatible properties. Due to its broad availability, pectin has become a prominent branch of the research and development of nature-based biomedical and healthcare areas. This article, therefore, aims to review the biological properties of pectin or pectic polysaccharides and summarize their biomedical applications in the emerging fields of drug delivery and tissue engineering, aiming at their expanding usage.

## 2. Properties of Pectic Polysaccharides

Pectin’s versatile properties allow it to be prospectively used in other applications, like medicine, as a carrier vehicle for drug delivery and a scaffold in tissue engineering or regenerative medicine.

### 2.1. Immunoregulatory Activity

The structural features of pectin provide a polysaccharide with biological activities such as immunomodulation. Immunomodulation is classified as a group of therapeutic interventions to regulate the immune system. Immunomodulators respond to the immune system by two different mechanisms: immunostimulation and immunosuppression. Immunosuppressive activity occurs on the backbone of pectin polysaccharides [15]. The structural changes in the galacturonic chain of the pectin control the macromolecule’s capacity to reduce immune reactivity [16]. The presence of a high quantity of galacturonic acid residues displays an increased immunosuppression activity. The amount of galacturonic acid residue fragments found on pectin determines the immunomodulatory effect. The injection of a glucan, zymosan, enables the pectin that contains more than 80% of the content of galacturonic acid residues to lower the production of macrophages. The polysaccharides of pectin that have 75% galacturonic acid residues or less do not reduce the gathering of macrophages stimulated by the injection of zymosan. Certain plants that produce pectin contain a significant percentage of galacturonic acid residues, while others do not. Plants with a high quantity of galacturonic acid residues include *Potamogeton natans* L., pond weeds that produce the pectin called *Potamogeton Anand*, and *Vaccinium oxycoccos* L. This cranberry plant produces the pectin called oxycoccusan. Plants that give rise to the pectins with lower than 75% galacturonic acid are those derived from Butomus, derived from Butomaceae, and Lemna, which emerged from Araceae. Table 1 shows pectin’s immunoregulatory activities.

### 2.2. Anti-Inflammatory Activity

Different degrees of methyl esterification affect the inflammatory properties of pectin. The various degrees of pectin methyl esterification play a role in determining the polysaccharide’s capacity to prevent the functional activity of white blood cells and leukocytes. In observing the influence of methyl esterification on pectin macromolecules, it is essential to analyze the makeup and characteristics of the pectin progenitor’s raw materials and the methods used to isolate the pectin. Table 2 summarizes the anti-inflammatory properties of pectin.

### 2.3. Antibacterial Activity

Biomedical applications of antimicrobial natural systems have gained much attention in recent years. Biodegradable natural products based on pectin, pectin-linoleate, pectin-oleate, and pectin palmitate were reported to inhibit the microbial effect on several bacterial strains, including *E. coli* and *S. aureus*. Table 3 shows the reported data on the antibacterial properties of pectin. Table 3 exhibits the antibacterial properties of various pectin.

### 2.4. Anticancer Activity of Pectin and Pectin-Based Composites

Effective cancer treatment, a significant global disease, is highly challenging. Even though there is a substantial advancement in surgery, gene therapy, immunotherapy, chemotherapy, and radiotherapy, the mortality rate due to metastatic cancer is still alarming. Drug resistance of cancer tumor cells and adverse side effects of chemotherapies have been considered the critical drawbacks of cancer treatment. Several in vitro and in vivo studies reported the anti-tumor activity of pectin that showed a decrease in tumor cell adhesion and proliferation and stimulation of cell apoptosis [37]. Table 4 displays the anticancer activity of pectin and other pectin-based composites.

## 3. Pectin for Drug Delivery Applications

**Figure 3:**
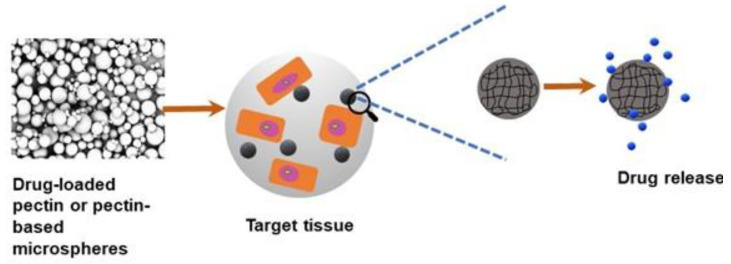
Schematic diagram of drug delivery approach using pectin or pectin-based microspheres, which release the drug at a controlled rate when injected/implanted to the target tissue in vivo.

Due to their excellent properties, such as biocompatibility, nontoxicity, flexibility in fabrication, and functionalization, natural polymer pectin hydrogels have gained extensive consideration in drug delivery applications. In the drug delivery system, the degradation rate of the hydrogel carrier is significant when delivering the active substance to the target site [54]. Using pectin within the drug delivery system has broadly been explored because pectin hydrogels can release drugs. Generally, researchers in the drug delivery system want the drug to be safely and efficiently immobilized or covalently attached to a biomaterial vehicle such as pectin [55]. Figure 3 shows the schematic diagram of a drug delivery system using pectin or pectin-based composites. Industrially, when integrated into a drug component, pectin is broadly utilized to treat radioactive isotopes and heavy metal poisoning [56]. Concerning heavy metals, pectin can act as a chelating agent by removing or preventing the interactions of toxic heavy metals within the human body, such as iron, copper, and mercury. When pectin polysaccharides interact with metal ions, esterification, and chelation occur according to the number of non-methyl-esterified galacturonosyl residues. Pectin molecules that are not esterified can form gels when surrounded by bivalent cations.

For this reason, when ionic crosslinks between galacturonan chains containing six or more adjacent residues increase, the metal binding of pectin molecules expands, and the degree of methyl esterification decreases [57]. The degree of esterification describes the percentage of galacturonic acids that react with methanol and are converted into an ester. There are generally two categories of pectin: high-methoxyl pectin and methoxyl pectin. When calcium surrounds the low-methoxyl pectin, the pectin gains the capacity to form gel because of the ionic crosslinking between the homogalacturonan chains. The egg-box mechanism is classified as one of the few gelation mechanisms that have been discovered [8]. In the egg-box process, six or more contiguous and non-esterified galacturonic residues are contained between each formed homogalacturonan chain within the calcium-crosslinked junction zones [8]. As a result of this interaction, an absorbent polymer network is formed [58]. The degree of methyl esterification of the galacturonosyl residues plays a crucial role in leading the gelation of the pectin polysaccharides and in determining the physical properties of the pectin. The pectin polysaccharides’ ability to form gels is one of the main reasons they are used within developed applications from various areas of the profession, such as physics, chemistry, biochemistry, biotechnology, cryobiology, and medicine [57].

Pectin has been used as a nourishing dietary component, and polysaccharides have also been used in drugs to treat diseases that develop from within the digestive system. Unmodified pectin is not digestible [59]. Within the human body’s digestive system, pectin activates the movement and peristalsis of the digestive system. Peristalsis is known as the wave-like movement that occurs for muscle contraction. Pectin can also cleanse the small intestine’s villi, and its gel properties enable it to improve the absorption of food intake and biologically stimulated materials. Pectin has been used as a drug delivery vehicle within the deliveries of colon-specific drugs and hydrogel-based drug delivery systems. Hydrogels generally consist of crosslinked, hydrophilic polymer chains that form three-dimensional networks [60]. In the hydrogel-based drug delivery system, pectin, as a drug delivery vehicle, can release the desired medication at a specific rate and area in the body [6]. In colon-specific delivery, the polymer can prevent specific drugs from traveling into the upper intestines, and instead, the drug is delivered into the colon. The polymer also can control the release of drugs at specific rates that are desired. Pectin’s magnitude of interaction with other diverse biopolymers leads to the production of new composite materials used in applications such as tissue engineering.

## 4. Pectin for Tissue Engineering Applications

**Figure 4:**
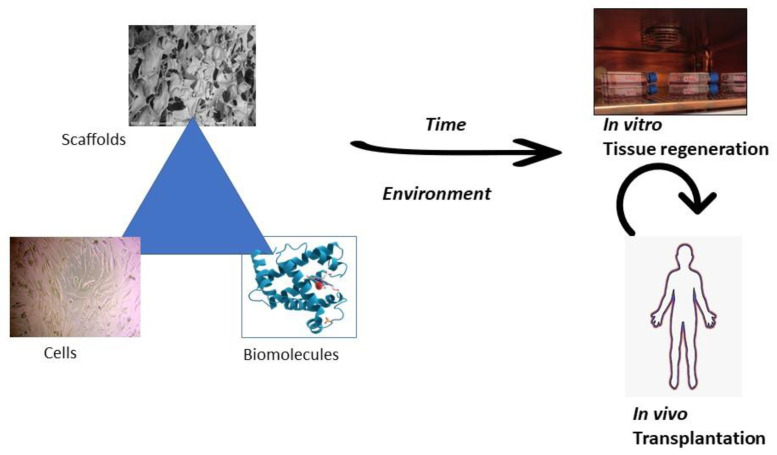
The fundamental tissue engineering strategy. With appropriate time and environment, the tissue engineering triad consisting of pectin-based scaffolds, autologous cells, and biomolecules contributes to tissue regeneration in vitro and later transplantation in the human body in vivo.

Tissue engineering is the strategy of regenerating damaged tissue using biomaterials (Figure 4). This application aims to use materials such as polymers that can mimic the natural cell formation and aid in the attachment, proliferation, and differentiation of cells [11]. In tissue engineering, damaged tissues are recovered when the body’s cells and the highly porous tissue scaffold are integrated as a template for forming the tissue’s new growth [12]. In tissue engineering, pectin generally acts as a matrix material [13]. Developing synthetic tissue engineering scaffolds that emerge from endogenous or transplanted parent cells gains the capacity to function based on environments that properly integrate signals that restore proper cellular processes.

Pectin has several advantages in tissue engineering, such as biodegradability, biocompatibility, low toxicity levels, antibacterial characteristics, and the polymer’s ability to promote controlled drug release. Hundreds of research articles on pectin’s applications in tissue engineering have been documented in the past ten years. Pectin is very attractive in tissue engineering because when it is fabricated into a tissue scaffold, it can control the release of drugs from the scaffold, which accelerates the local regenerative activity [61]. Pectin can also be modified in numerous ways with compounds and other biopolymers. When adjusting the pectin biomaterial for applications such as tissue engineering, it is essential to use biopolymers and compounds that interact well with it and promote the overall efficiency of the composite in combination with it. There are two main ways that interactions can occur between polysaccharides and compounds: (1) repulsion by steric exclusion and (2) attraction between the molecules. When pectin is modified with oligopeptide-arginine-glycine-aspartic, the fabricated composite can improve preosteoblast generation via cell adhesion and differentiation better than pectin alone [52]. Recently, pectin constructed with chitosan has been explored in tissue engineering as a scaffold to regenerate damaged tissue such as bone and skin. Porous pectin-based tissue scaffolds are generally fabricated using standardized techniques such as freeze-drying. Combined, chitosan and pectin create a polyelectrolyte complex that results in a smart scaffold that has improved mechanical resistance, porous microstructures, swelling capacity, stabilized crosslinking, and biocompatibility [62].

Modified pectin has even been explored in regenerating tissue parts such as the ear and nose. A 3D anatomical-shaped scaffold of the ear and nose was developed in a study using the composite of pectin and (3-glycidyloxypropyl) trimethoxysilone (pectin-GpTMS). The pectin–GpTMS greatly benefits as a biomaterial in mimicking different tissues for patient-specific scaffolds [63]. The diverse abilities that pectin has are why it is widely used globally in various biomedical applications. Table 5 demonstrates the application of pectin systems in tissue engineering strategy.

## 5. Conclusions and Future Prospects

This review summarizes the important biological properties and recent development of pectin and pectin-based composites in biomedical applications. Pectin and pectin-based composites are attractive for biomedical applications due to their nontoxicity, biocompatibility, biodegradability, anti-tumor, antibacterial, and anticancer characteristics. These materials also have excellent chemical reactivity and emulsification properties, which make them widely used and an advanced candidate for drug delivery and tissue engineering applications. Developing functionalized scaffolds for bone tissue, skin, biological valves, and injectable scaffolds is being explored, promising natural macromolecular materials for medical applications. However, further investigations are needed to explore insights into the roles of bioactivity of pectin in vivo. A lack of available research on the mechanisms of pectin’s anticancer protection mechanisms, as well as clinical trials, has restricted pectin’s application in medicine and drug development. A combination of in vitro and in vivo degradation kinetics, information on digested products, and the mechanisms of actions could further illuminate how pectin can be further technologically explored to expand its applications in the clinical setting of the biomedical and tissue engineering field.

## Figures and Tables

**Table 1:** Pectin’s immunoregulatory activities: source and mechanism of action.

Pectin Source	Uses and Mechanism of Action	Reference
i. Lemon Pectin	The physical-chemical characteristics of lemon pectin, for example, the degree of methyl esterification and the extent of polymerization, influence the immunostimulatory properties. It is significantly essential to utilize pectins to improve immune response.	[17,18]
ii. *Sumbuci floss* or elderflower	Used to heal various diseases linked with the immune system, for example, influenza, chill, or pyrexia. Extracts from S. nigra flowers have stimulation effects on macrophages. In vitro studies reported that the biological activity of rhamnogalacturonan I (RG-1) comprising polysaccharides of elderflowers contributes to higher immunomodulation activity and enhanced macrophage-stimulating effects.	[1,19]
iii. Tomato Pectin	Pectic oligosaccharides in sour raw tomatoes demonstrated potential as an anticancer on a gastric cancer cell line in vitro.	[20]
iv. *Lycium ruthenium*	Polysaccharides in *L. ruthenium* suppressed proinflammatory cytokines in lipopolysaccharide-stimulated macrophages and exhibited antifatigue, antioxidation, and hypoglycemic activity.	[21,22]

**Table 2:** Anti-inflammatory properties of pectin.

Pectin Source	Mechanism of Action	Reference
i. Star fruit (*Averrhoa carambola* L.)	In vivo, the study reported that the polysaccharides from starfruit exhibited antinociceptive and anti-inflammatory properties and were beneficial for controlling inflammatory pain.	[23,24]
ii. *Suaeda fruiticosa* (L.) Forssk	Polysaccharides, phenolic compounds, and bioactive flavonoids from *S. fruticose*, comprising free radical scavenging and lipid peroxidation, function as an anti-inflammatory agent and analgesic or antioxidant.	[25,26]
iii. Citrus pectin	An in vivo study demonstrated that low methyl-esterified pectin from citrus fruits inhibited systemic and local inflammation, whereas a high degree of esterification inhibited intestinal inflammation.	[15,27]
iv. Sweet pepper fruits	Both native and modified pectin possessed the inherent activity to control THP-1 macrophages. Due to the availability of lipopolysaccharides, anti-inflammatory properties occur by inhibiting proinflammatory and promoting anti-inflammatory cytokines.	[28,29]

**Table 3:** Antibacterial properties of pectin-based composite materials.

Pectin-Based System	Mechanism of Action	Reference
i. Citrus pectin-coated Ag nanoparticles (NPs)	Citrus pectin-coated Ag NPs exhibited great antibacterial activities toward Gram-negative *E. coli* and Gram-positive *S. Aureus*.	[30]
ii. Pectin–cadmium sulfide nanocomposite (Pc/CSNC); pectin–zirconium (IV) silicophosphate nanocomposite (Pc/ZSPNC)	Pc/CSNC exhibited a significant effect of antibacterial activity against *E. coli.* PC/ZSPNC showed substantial antibacterial activity towards *E. coli* and *S. aureus.*	[31,32]
iii. Citrus pectin–MgO nanocomposites	Pectin–MgO showed significant antibacterial activity against clinical pathogens lactobacillus and Bacillus subtills.	[33]
iv. Pectin/lysozymes layer-by-layer nanofibrous mats	Pectin/lysosome nanofibrous mats exhibited significant antibacterial effects against *E. coli* and *S. aureus*.	[34]
v. Essential oils (EOs)/Pectin nanoemulsion	EOs/Pectin nanoemulsion exhibited antibacterial activity towards *E. coli* and *L. innocua* populations.	[35,36]

**Table 4:** Anticancer activity of pectin and pectin-based composites.

Pectin Source or Pectin-Based System	Target Cancer Cell Line	Mechanism of Action	Reference
Pectin from potato	Human colon cancer HT-29 cells	Rhamnogalacturonan (RG)-I domain-rich potato pectin showed the inhibitory effect of HT-29 cell proliferation in vitro.	[38,39]
Pectin from sugar beet	Human colon cancer cell lines (HT-29 and DLD-1)	An in vitro study reported that the pectin from sugar beet exhibited antiproliferative activity toward colon cancer cells—alkali-treated sugar beet pectin extract induced apoptosis.	[40]
Pectin from sweet potato	Human colon cancer HT-29 cells	Sweet potato pectin modified by ultrasonication inhibited HT-29 cell proliferation and induced apoptosis in vitro.	[41]
Pectin from apple	Breast cancer cells 4T1	Pectic acid from apple pectin inhibited 4T1 breast cancer cell growth, reduced cell attachment, and induced apoptosis in vitro. In vivo, results exhibited that pectic acid inhibited tumor progression and increased apoptosis cell number.	[42]
Citrus pectin	Liver hepatocellular carcinoma cells HepG2 and adenocarcinoma human alveolar basal epithelial cells A549	Citrus pectin (heat-modified) induced classical apoptosis and indicated the activation of autophagy in both HepG2 and A549 cancer cell lines.	[43]
Pectin from papaya	Colon cancer cell, prostate cancer cell	Papaya pectin extracted from intermediate ripening phases significantly decreased cell viability and induced necroptosis in cancer cell lines in vitro.	[44]
Pectin–curcumin	Breast and hepatic cervical cancer cells	The pectin–curcumin complex had better inhibitory activity against cancer cells than only curcumin due to the increased stability and solubility of the composites.	[45,46]
Pectic polysaccharide/Selenium	Adenocarcinomas human alveolar basal epithelial cells	Pectic polysaccharides/selenium showed a higher inhibiting capacity for cell migration and initiated cell apoptosis than the original pectin polysaccharides.	[47]
Pectin-polyvinyl pyrrolidone–curcumin	Lung cancer cells A549	Pectin–polyvinyl pyrrolidone–curcumin particulates showed increased anti-tumor effects than curcumin alone.	[48]
Pectin/silver (Ag) nanocomposites	Epithelial human breast cancer cell line MDA-MB-231	Pectin/Ag nanocomposites showed a significantly high inhibitory effect on breast cancer cell proliferation.	[49]
Pectin/gold nanoparticles	Mammary adenocarcinoma	Pectin/gold nanoparticles induced apoptosis and decreased the viability of the cancer cells.	[50]
Citrus pectin/Zn nanoparticles	Ehrlich ascites carcinoma and human colon adenocarcinoma	The citrus pectin/Zn nanoparticles showed anticancer properties by influencing cancer cell cytotoxicity.	[51]
Pectin/chitosan	Human colon cancer HT-29 cells	Pectin/chitosan composites exhibited antiproliferative effects on cancer cells but no cytotoxic effects on normal cells.	[52]
Pectin aldehyde/ poly(N-isopropyl acrylamide-*stat*-acyl hydrazide) P(NIPAM-*stat*-AH)	Colon carcinoma cells CT26	In vivo, the study revealed that the self-healing and injectable composites had the potential for anticancer therapy.	[53]

**Table 5:** A summary of several pectin systems in a tissue engineering approach.

Pectin Systems	Method	Application	References
Low-methoxyl citrus pectin	UV photocrosslinking with peptide crosslinkers (cell-degradable) and adhesive ligands (integrin-specific); lyophilization	Skin tissue engineering	[7]
Sugar beet pectin (SBP) crosslinked by visible light	Applying 405 nm visible light in the presence of tris(bipyridine)ruthenium (II) chloride hexahydrate and sodium persulfate, rapid hydrogenation of SBP was obtained; 3D hydrogel constructs were obtained using 3D bioprinting	Promising for liver and other soft tissue engineering	[64]
Citrus peel’s pectin crosslinked with (3glycidyloxypropyl) trimethoxysilane (GPTMS)	Freeze-drying or 3D bioprinting	Various tissue regeneration	[63]
Pectin/chitin/nano CaCO_3_	Lyophilization	Bone regeneration	[13]
Pectin/chitosan	Freeze-drying	Bone tissue engineering	[5]
Pectin/strontium/hydroxyapatite	Solution-based chemical technique	Bone regeneration	[65]
Collagen/polyurethane/pectin	Semi-interpenetration process	Bone regeneration	[66]
Pectin/PVA	Freezing–thawing	Bone regeneration	[67]
Poly(L-lactide-co-ε-caprolactone) (PLCA)/pectin	Scaffolds functionalized with pectin	In vitro and in vivo bone regeneration	[68]

## Data Availability

No new data were created.
